# Queuing Time Prediction Using WiFi Positioning Data in an Indoor Scenario

**DOI:** 10.3390/s16111958

**Published:** 2016-11-22

**Authors:** Hua Shu, Ci Song, Tao Pei, Lianming Xu, Yang Ou, Libin Zhang, Tao Li

**Affiliations:** 1 State Key Laboratory of Resources and Environmental Information System, Institute of Geographic Sciences and Natural Resources Research, CAS, Beijing 100101, China; shuh@lreis.ac.cn (H.S.); songc@lreis.ac.cn (C.S.); 2 University of Chinese Academy of Sciences, Beijing 100049, China; 3 RTmap Science and Technology Ltd., Beijing 100093, China; xulianming@rtmap.com (L.X.); ouyang@rtmap.com (Y.O.); 4 Information Technology Department, Beijing Capital International Airport Co., Ltd., Beijing 100621, China; zhanglb@bcia.com.cn (L.Z.); lit2@bcia.com.cn (T.L.)

**Keywords:** indoor queuing time, WiFi positioning, trajectory, mobile, time series analysis

## Abstract

Queuing is common in urban public places. Automatically monitoring and predicting queuing time can not only help individuals to reduce their wait time and alleviate anxiety but also help managers to allocate resources more efficiently and enhance their ability to address emergencies. This paper proposes a novel method to estimate and predict queuing time in indoor environments based on WiFi positioning data. First, we use a series of parameters to identify the trajectories that can be used as representatives of queuing time. Next, we divide the day into equal time slices and estimate individuals’ average queuing time during specific time slices. Finally, we build a nonstandard autoregressive (NAR) model trained using the previous day’s WiFi estimation results and actual queuing time to predict the queuing time in the upcoming time slice. A case study comparing two other time series analysis models shows that the NAR model has better precision. Random topological errors caused by the drift phenomenon of WiFi positioning technology (locations determined by a WiFi positioning system may drift accidently) and systematic topological errors caused by the positioning system are the main factors that affect the estimation precision. Therefore, we optimize the deployment strategy during the positioning system deployment phase and propose a drift ratio parameter pertaining to the trajectory screening phase to alleviate the impact of topological errors and improve estimates. The WiFi positioning data from an eight-day case study conducted at the T3-C entrance of Beijing Capital International Airport show that the mean absolute estimation error is 147 s, which is approximately 26.92% of the actual queuing time. For predictions using the NAR model, the proportion is approximately 27.49%. The theoretical predictions and the empirical case study indicate that the NAR model is an effective method to estimate and predict queuing time in indoor public areas.

## 1. Introduction

Queuing is a pervasive phenomenon in urban public places, such as supermarkets, restaurants, banks, transportation terminals and amusement parks [1]. Long-duration queuing reflects the imbalance between the supply and demand of public services, which not only takes up many individuals’ time and leads to negative experiences but also burdens management and reduces the capacity of such services. Furthermore, research has shown that, in addition to the length of waiting time [2], an important factor affecting individuals’ service satisfaction is whether the waiting time is foreseeable [3]. The ability to estimate and predict queuing time reduces the queuing time through self-planning and relieve anxiety. Moreover, it promotes the responsible use of public resources and enhances the level of management and public security. Therefore, real-time queuing time measurement and prediction in public places is of significant importance to the community.

Automatic queue-monitoring techniques are fundamental to real-time queuing time measurement and prediction. In this study, we focus on queuing time in indoor public areas. The indoor queuing activity monitoring technologies used in previous studies mainly include video surveillance, radio frequency identification (RFID), Bluetooth, accelerometers, and WiFi (wireless fidelity), which will be briefly introduced below.

- *Video Surveillance*. By employing video content analytics to detect the number of people queuing and to estimate the time needed to leave the queue [4,5,6], the queuing time of an individual who is just entering a queue can be estimated [7] using the following equation:
  (1) $$T_{avg}\approx \left(\int_{0}^{\infty }xp_{N}\left(x\right)dx\right)\left(\int_{0}^{\infty }sp_{s}\left(s\right)ds\right)$$
  where $p_{N}\left(x\right)$ is the density function of the number of people in the queue, and $p_{s}\left(s\right)$ is the density function of service time. However, video surveillance may compromise individual privacy, and in large-scale areas where the number of people queuing is large and the queue structure is complex, people counting may have poor accuracy.
- *Radio Frequency Identification (RFID)*. By using the protocol for communication between user tags and fixed readers near the counter [8]), RFID readers can detect the existence of nearby tags. The queuing parameters can be tracked based on the number of detected tags and signal strength [9]. However, RFID is not a commonly used technology, so extra hardware and individuals’ active participation are required, which may not be accepted by managers and users.
- *Bluetooth*. An individual’s position with time stamps can be acquired using Bluetooth localization [10,11] with positioning methods such as proximity, trilateration, and fingerprinting. Then, the queue entry and exit times [12] can be determined based on the relationship between a queue process and a spatial location to estimate the queuing time. Queuing time estimation using Bluetooth is highly accurate. However, special equipment is needed to detect the Bluetooth signals from the users’ mobile devices.
- *Accelerometer*. The accelerometers built into mobile devices can be used to record a user’s continuous movement [13] (based on acceleration parameters). An individual’s movement while queuing is essentially standing-walking-standing [14], which is reflected by fluctuation in the acceleration curve. Using pattern recognition [15], the queuing period can be determined based on moving status; thus, queuing time be calculated. However, users must install an application on their mobile device to continuously upload their moving status data.
  All of the techniques mentioned above can be used to monitor queuing activity and automatically estimate queuing parameters. However, in practice, these techniques have three main deficiencies: they require extra equipment to track queuing activity, they require active participation, and they may compromise individuals’ privacy.
- *WiFi*. WiFi is mainly used for wireless Internet access equipment in public places and private homes. In addition to the communication function, WiFi is widely used in indoor navigation [16,17], targeted advertisements [18,19], and behavior analysis [20,21]. However, little research has been conducted on the use of WiFi for queuing time monitoring. This technique overcomes the disadvantages of other approaches. In previous studies, users were required to install an app on their mobile device to communicate signal information with the backend. WiFi queuing time monitoring methods can be divided into two categories: (1) signal threshold judgment; and (2) signal feature analysis.

### 1.1. Signal Threshold Judgment

Each WiFi access point (AP) has a unique broadcast service set identifier (BSSID). An AP generates beacons to broadcast its existence. If a mobile device enters the signal transmitting range of an AP and the signal strength exceeds a certain threshold, it will track the beacons of an AP located in a specific indoor location, and the user is considered to have commenced queuing in this location. When the beacons are no longer being tracked by the mobile device, the user’s queuing process has come to an end. Thus, the queuing time of a user is estimated based on time stamps that indicate when the beacon tracking by the mobile device begins and ends. Because the continuous presence of WiFi sensing beacons consumes energy, location sensing and behavior analysis from other technologies can be used to (de)activate the sensing process [15,22]. Although this approach only requires WiFi and a mobile device to monitor queuing time, the spatial resolution is too coarse to accurately determine the entry and exit time of a queue.

### 1.2. Signal Feature Analysis

A typical service process represented on the time—received signal strength (RSS) curve can be divided into three phases: the queuing phase, the service phase and the leaving phase. To track queuing, we can deploy a WiFi monitor near the queue start and destination. The WiFi monitor can record the RSS of an individual’s mobile device. From this measurement, the time-signal intensity curve can be derived. RSS usually decreases with increasing distance from a mobile device to the WiFi monitor. As the people queuing move toward the counter, the signal strength increases. During the service period, the signal strength tends to be the strongest and remains stable. When the service is complete and people leave, the signal strength drops off dramatically. Thus, the queuing time and service time can be determined from the signal strength [23,24,25]. The advantage of this method is that it requires only one WiFi monitor to act as an active sensor and the service time can be estimated accurately. However, this method has difficulty recognizing the starting time of a queue, and it requires users to install an app on their mobile devices to improve the frequency of sending data packets to WiFi APs to produce detailed time-signal strength curves.

Compared with previous WiFi-based solutions for detecting queuing time, WiFi positioning technology that captures people’s locations with time stamps has become the state-of-the-art for queuing time estimation and prediction in indoor scenarios. However, most application scenarios are restricted by site-specific environmental, security and management requirements, so the number and deployment locations of APs are often limited. Thus, the positioning accuracy usually cannot be optimized, and sometimes may be quite poor. Therefore, in our research, we propose a method that uses topological relation (contained or disjoint) judgment between WiFi positioning locations and the queue zone to estimate individuals’ queuing time, that is, during the positioning system deployment phase, we give priority to the topological correctness of positioning (the topological relation between an individual’s positioning location and the queue zone remains consistent with their current topological relation) rather than its accuracy. To achieve this, three principles are followed: (1) some of the APs in a queue zone are deployed close to and surrounding the border of the queue zone; (2) the signals of the border APs must be directed toward the inside of the queue zone; and (3) APs outside the queue zone must not be too close to the border of the queue zone and have relatively low signal strength. Using these principles, we can obtain reliable topological accuracy, and the queuing time can be determined by WiFi positioning data with acceptable precision. 

For queuing time prediction, time series analysis, queue theory and traffic flow theory are common. Time series uses mathematical methods to extract the trends, seasonality and other features of a data sequence; or to smooth the data sequence by combining information from present and previous observations. Previous studies have shown that exponential smoothing [22,26,27], Holt-Winters [22,28,29] and seasonal trend decomposition based on loess (STL [30,31,32]) can achieve good accuracy for queuing time forecasting. Queue theory [33,34] and traffic flow theory [35,36] use statistical and physical models to estimate queuing parameters or to model the traffic flow process and predict future queuing parameters. However, they are not applicable in this work, because parameters such as speed, density, and flow in traffic flow theory and queue discipline, arrival rate, and service rate in queue theory are required to model the queuing process, while the collected data are spatial location data with time stamps [22], from which these parameters are difficult to obtain.

The advantages of our method using WiFi positioning data to estimate and predict queuing time in indoor scenarios are as follows:

(1) The method for estimating the queuing time from topological relation judgment between individuals’ trajectories and the queue zone is robust in realistic application scenarios.
(2) No special sensors are needed to track people’s queuing activity, because WiFi and mobile devices are ubiquitous in modern life.
(3) Users are passive because no active cooperation is required and no additional apps need to be installed on their mobile devices.
(4) No consideration for the length or shape of the queue is required because queuing time only depends on the time stamps when an individual enters and leaves a queue zone.

The remainder of this paper is organized as follows. In the following section, we provide an overview of WiFi positioning technology, and introduce how to deploy the positioning system and determine queuing time using positioning data. Then, we explain the prediction models (i.e., NAR, Holt-Winters, and STL-ARIMA) based on time series analysis theory. Next, we validate our positioning precision and estimation model in a real indoor scenario, and present a case study conducted at the T3-C entrance (one entrance of Terminal T3) of the Beijing Capital International Airport. We then discuss the practicability of this research and factors that affect the accuracy of the estimation and prediction results. Finally, we present our conclusions and suggest areas for future work.

## 2. Theory and Methodological Framework

The queuing time prediction method in this research includes two main parts, i.e., hardware deployment and the algorithm. In the hardware deployment part, in consideration of the high maneuverability and portability, we use a trilateration method instead of fingerprinting (fingerprinting has relatively high positioning accuracy but it is hard to establish fingerprint databases in large or crowded public places and it is difficult to upgrade the system [37]). In order that the spatial topology relation between spatial locations and a queue zone can be used for queuing time determination, we installed metal baffle plates for the border APs to improve the topological correctness. To identify relatively reliable samples as representatives to estimate queuing time, in the algorithmic part, we set four parameters in the estimation model. As our estimation results did not show obvious periodic features and consistent trends, we used a nonstandard autoregressive (NAR) model without considering the seasonality for prediction. To enable comparison with methods considering seasonality and trends in previous queuing time prediction studies, two other models in time series analysis (i.e., Holt-Winters and STL-ARIMA) are also tested.

### 2.1. WiFi Positioning Technology

In addition to communication functions [38], WiFi is used as a location service based on indoor positioning techniques, which compensates for the shortcomings of GPS positioning, whose signal is too weak to be received indoors. There are three broad classes of location techniques that are used for WiFi positioning—triangulation, fingerprinting and proximity [39,40]. In this paper, we use trilateration, which is a triangulation technique, to track individuals’ locations. The key of this approach lies in the measurement of distances between target mobile devices and APs using radio waves. The radio wave propagation model is given as follows [17,41]):

(2) Pr(d)=Pr(d0)−10αlog(dd0)+ξ

where $P_{r}\left(d\right)$ is the signal strength received by the mobile device at distance d from the AP, $d_{0}$ is a known distance from the AP, α is the propagation loss coefficient, and ξ is a random variable that is unrelated to the propagation distance.

**Figure 1:**
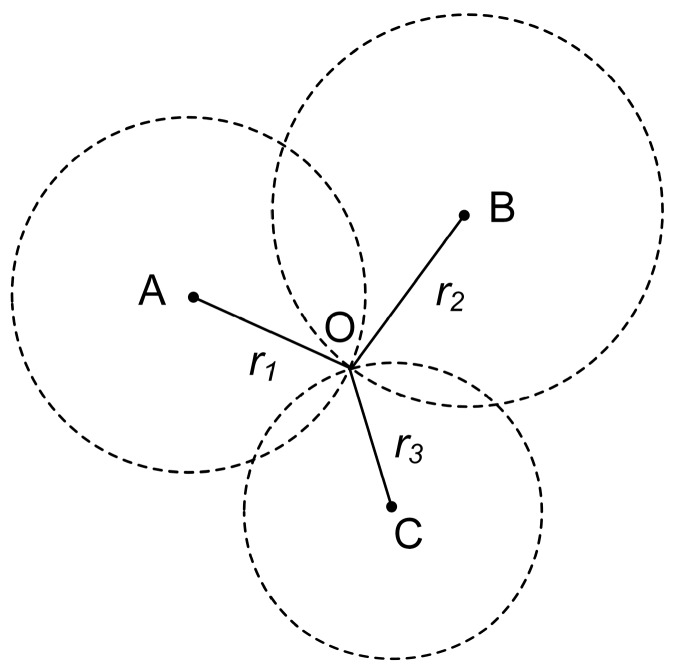
Theoretical map of the trilateration positioning method.

Figure 1 shows the location method of trilateration. The locations of the APs are represented by points A, B and C, of which the coordinates are measured in advance. The distances from the location of the target device (O) to the APs are represented by $r_{1}$, $r_{2}$ and $r_{3}$, which can be measured using the propagation model (model 1). Then, the coordinates of the target device are calculated using the least squares algorithm [42].

### 2.2. Hardware Deployment Principles

To guarantee topological correctness, as mentioned in Section 1, during the hardware deployment phase, three principles must be followed. For the first principle, according to reference [43], the signal strength error is greater when mobile devices are too far from the APs, resulting in increased positioning error. The positioning error near the inside border of a queue zone is the main factor that affects topological correctness. Therefore, a feasible solution is to ensure that some of the APs inside the queue zone are deployed close to and surrounding the border. For the second principle, as the location from our positioning algorithm is calculated by at least three APs that provide the strongest signal, we adjust the direction of the antennas and place metal baffle plates for the border APs to reduce the signal strength on the outside of the queue zone. Thus, when a user steps outside the queue zone, his (her) device will receive weak signal from the border APs and his (her) position is more likely to be located outside the queue zone. For the third principle, a user near the inside border of a queue zone must remain for a relatively long time to wait his (her) turn rather than stay near the outside border while rapidly leaving. To avoid long-term topological error (inside-outside error in Section 3.2), a user’s device should receive a relatively low strength signal from the outside APs than the inside APs while waiting inside the queue zone. Therefore, the outside APs must not be too close to the border of the queue zone [17] and have relatively low signal strength.

### 2.3. Queuing Time Estimation Based on Positioning Data

To estimate a crowd’s average queuing time, the optimal solution is to determine the queuing time of each individual. However, the time intervals of the trajectory points from our passive positioning system range from five seconds to several minutes, which results in incomplete queuing process records. In addition, the sample errors are not negligible. Therefore, sample reliability cannot be neglected, that is, more samples may not produce more accurate results. Therefore, similar to using a buoy to estimate the flow velocity of a river reach, a feasible solution is to identify relatively reliable samples acting as representatives [14] for queuing time estimation. To achieve this goal, when determining an individual’s queuing time, four parameters (m, n, k, and r) of the trajectory are used. In practical applications, these parameters need to be tuned according to the site environment and hardware deployment. The estimation process is divided into three steps: (1) queuing process recognition; (2) parameter setting and queuing time determination; and (3) queuing time estimation.

#### 2.3.1. Queuing Process Recognition

To capture the queuing process of an individual using positioning data, we designate in advance a physical or virtual fence with clear boundaries to serve as the queue zone. An integrated trajectory for queue process capture can be divided into three parts: trajectory before entering the queue zone, trajectory in the queue zone and trajectory after leaving the queue zone. The time intervals between these three parts must be reasonably small. Additionally, the origin and destination points of a trajectory must be outside of the queue zone and on different sides, and the direction from the origin to destination must be in accordance with the heading direction of the queue.

#### 2.3.2. Parameter Setting and Queuing Time Determination

**Figure 2:**
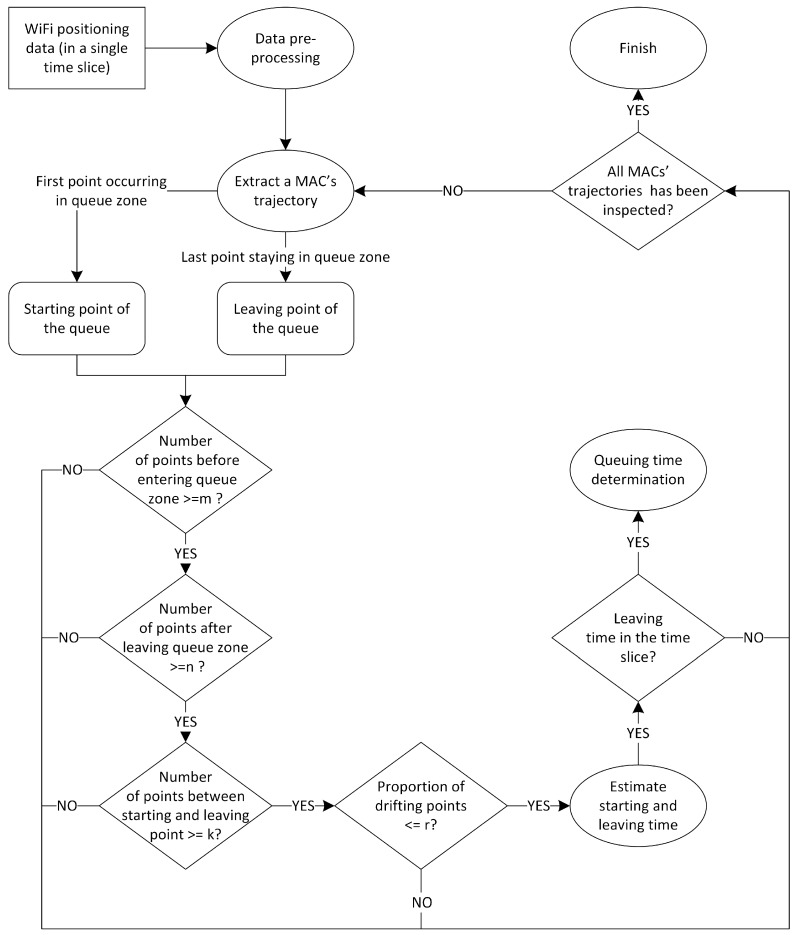
Process flow of the queuing time determination model.

The starting and leaving times of a queuing process are the keys to an individual’s queuing time determination. Topological relations between a queue zone and points on trajectories with time stamps can help to discern the starting and leaving points of a queue process to estimate the queuing time. Figure 2 shows the queuing determination process of individuals. As mentioned above, during this process, four parameters (m, n, k, and r) are used to discern relatively reliable trajectories.

**Figure 3:**
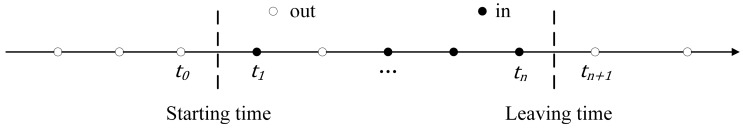
Queuing sequence of an individual in the time domain.

Figure 3 shows a queuing process in the time domain. The first point when an individual enters the queue zone is regarded as the starting point (point at time $t_{1}$), and the last point before leaving the queue zone is the leaving point (point at time $t_{n}$). The locations of the points at times $t_{1}$ and $t_{n}$ are taken as the starting and leaving points because all points before time $t_{1}$ and after time $t_{n}$ are outside the queue zone. These adjacent points indicate that the chance that they are drift points from the inside of queue zone is small. The frequency of packet delivery is affected by the WiFi module status of the mobile devices (e.g., “on” but not connected to WiFi APs, and “connected” but not using the network traffic). This is especially the case when the WiFi module is switched on but not connected to the WiFi APs, the device is only used intermittently, or the device has not been used during the queuing process. In these cases, the packet delivery frequency may be low and irregular. Thus, the points on the trajectory will be sparse and uneven. Therefore, the parameters defining the absolute number of points in each part of the trajectory are set to ensure the trajectory is eligible for analysis, i.e., at least m points before entering the queue zone, n points after leaving the queue zone and k points from $t_{1}$ to $t_{n}$ (including points at times $t_{1}$ and $t_{n}$). Moreover, the locations determined by WiFi positioning may drift accidently (“drift phenomenon” or “drift weakness” hereafter, e.g., an individual inside a queue zone having positioning location outside the queue zone), which may lead to topological errors. To alleviate the impact of inside-outside drift phenomena (refer to Section 3.2), which causes obvious estimation inaccuracies, a parameter called the drift ratio (r) is required. The trajectory can be used to estimate the queuing time only if the proportion of drift points (empty circles between $t_{1}$ and $t_{n}$ in Figure 3) between time stamps $t_{1}$ and $t_{n}$ is less than the threshold. For available trajectories, we approximate the starting and leaving times of a queuing process as $\left(t_{0}+t_{1}\right)/2$ and $\left(t_{n}+t_{n+1}\right)/2$, respectively, and the total queuing time as $\left(t_{n}+t_{n+1}\right)/2-\left(t_{0}+t_{1}\right)/2$.

#### 2.3.3. Queuing Time Estimation

**Figure 4:**
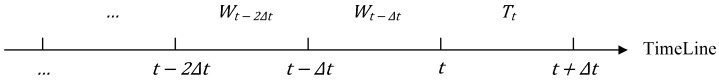
Time slicing for queuing time estimation and prediction.

To reflect the queuing time variation over time, one day is divided into equal time slices. Then, individuals’ queuing times are divided into specific slices. As shown in Figure 4, the time of a day is sliced by Δt. Let $t_{out}=\left(t_{n}+t_{n+1}\right)/2$ (see Section 2.3.2) be the time when an individual leaves the queue zone; if $t_{out}\in \left[t,t+\Delta t\right)$, the queuing time of the individual is aggregated to slice t to t+Δt. Let $\left\{w_{t}^{i}\right\}$ be the set of individuals’ queuing times determined by WiFi positioning data acting as representatives in time slice t to t+Δt, then $W_{t}=\overset{N}{\underset{i=1}{\sum }}w_{t}^{i}/N$ (where N is the number of individuals) is the WiFi-based average queuing time estimation of the slice.

### 2.4. Queuing Time Prediction

#### 2.4.1. Nonstandard Autoregressive

Autoregressive (AR) is a frequently used time series prediction model that assumes the present observation has a linear relation with previous observations. Generally, the notation AR(p) denotes an autoregressive model of order p. In this study, we use a nonstandard AR model, denoting as NAR(p):

(3) $$P_{t}=c+\overset{p}{\underset{i=1}{\sum }}\varphi_{i}W_{t-i\Delta t}+\varepsilon_{t}$$

where $P_{t}$ is the prediction of the actual queuing time at time slice t to t+Δt, c is a constant, $\varphi_{i}$ is the regression coefficient and $\varepsilon_{t}$ is the random error. In contrast to the general AR model, the parameters of NAR are trained using the previous observations (WiFi estimations) and the present ground truth (security system records).

#### 2.4.2. Holt-Winters

Let $\left\{W_{t}\right\}$ be the sequence of the crowds’ average queuing time based on the WiFi estimation from time t to t+Δt, with a seasonal variation of length L. The additive Holt-Winters prediction method comprises three equations. $\left\{s_{t}\right\}$ represents the sequence of smoothed values contributing as level, $\left\{b_{t}\right\}$ represents the sequence of the linear trend, $\left\{c_{t}\right\}$ is the sequence of the seasonal correction factors, and ∂, β and γ are the smoothing parameters. Then, the components of the additive Holt-Winters are:

(4) $$s_{t}=\alpha \left(W_{t}-c_{t-L\Delta t}\right)+\left(1-\alpha \right)\left(s_{t-\Delta t}+b_{t-\Delta t}\right)$$

(5) $$b_{t}=\beta \left(s_{t}-s_{t-\Delta t}\right)+\left(1-\beta \right)b_{t-\Delta t}$$

(6) $$c_{t}=\gamma \left(W_{t}-s_{t-\Delta t}-b_{t-\Delta t}\right)+\left(1-\gamma \right)c_{t-L\Delta t}$$

(7) $$P_{t+m\Delta t}=s_{t}+mb_{t}+c_{t-\left(L-1-m_{L}^{*}\right)\Delta t}$$

where Equation (4) is a weighted average of the seasonally adjusted estimation ($W_{t}-c_{t-L\Delta t}$) and the non-seasonal forecast $\left(s_{t-\Delta t}+b_{t-\Delta t}\right)$ for time slice t to t+Δt. Equation (5) is the trend which is actually Holt’s linear method [28]. Equation (6) is a weighted average between the current seasonal index ($W_{t}-s_{t-\Delta t}-b_{t-\Delta t}$) and the seasonal index of the same time slice of the previous season ($c_{t-L\Delta t}$). Equation (7) is the prediction function, where m is the step length of the prediction, $P_{t+m\Delta t}$ is the queuing time prediction of time slice t+mΔt to t+(m+1)Δt, and $m_{L}^{*}=\left[\left(m-1\right)\;mod\;L\right]$, which ensures the seasonal factors for prediction come from the most recent period.

#### 2.4.3. Seasonal Trend Decomposition with Autoregressive Integrated Moving Average (STL-ARIMA)

Seasonal trend decomposition (STL) is a statistical method that deconstructs a time series into several components, namely, trend ($TR_{t}$), seasonality ($S_{t}$) and remainder ($R_{t}$). STL is used to decompose time series, while Loess is used to estimate nonlinear relationship. The additive STL model adopted in this work is as follows:

(8) $$W_{t}=TR_{t}+S_{t}+R_{t}$$

where $W_{t}$ is a sequence of WiFi-based average queuing time estimations, $TR_{t}$ is the long-term increasing or decreasing trend in the sequence, $S_{t}$ is the regular fluctuation of seasonal factors and seasonality is fixed and has known period for a given sequence of periodic features. $R_{t}$ is the remainder of the sequence after components $TR_{t}$ and $R_{t}$ are removed and can be seen as noise.

The STL is a filtering process for a time series. The ARIMA model is used for prediction based on reconstructed a sequence from STL [31,44] (i.e., STL-ARIMA prediction model). In the ARIMA model, the variation of the observation sequence over time is regarded as random, and a statistical model is constructed to model the features of the sequence based on processes, such as AR and Moving Average (MA). The notation of the model is ARIMA(p,d,q), where p is the order of the autoregressive, d is the number of differences required for stationarity, and q is the order of the moving-average process. Readers can refer to Box and Jenkins et al. [44] for more details.

## 3. Methodology Validation

Queuing time determination and estimation based on WiFi positioning data is the basis of further prediction. Therefore, in this section, we only validate the effectiveness of hardware deployment principles and the accuracy of a crowd’s average queuing time estimation based on WiFi positioning data. The queuing time prediction by time series models mentioned in Section 2 is presented in the case study section.

### 3.1. Positioning System Deployment

**Figure 5:**
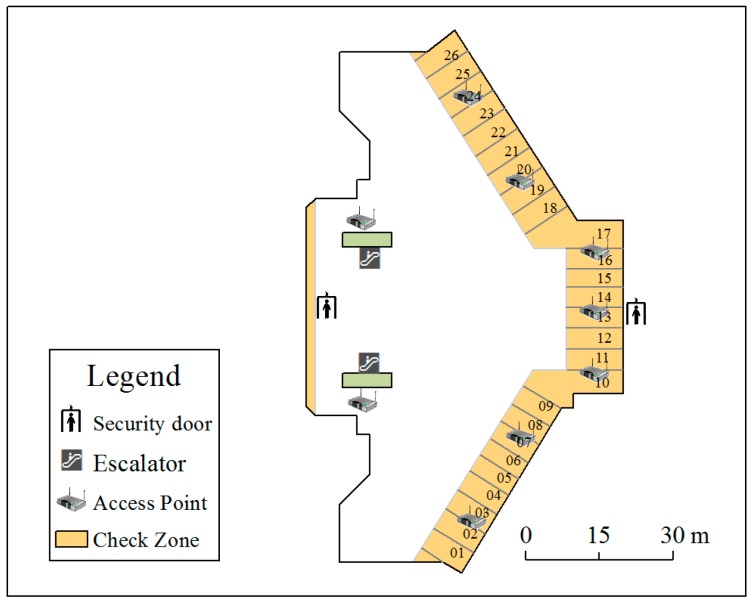
General overview of the queue zone and the locations of the APs.

To validate our method, we designed a queuing time monitoring system at the T3-C entrance of Beijing Capital International Airport. The trilateration method was chosen for the positioning technique. In the deployment phase of APs, we aimed to minimize the systematic errors. Le Dortz et al. [45] verified that using more APs can help to improve the stability of WiFi positioning. Therefore, a greater number of APs improves the topological correctness. Regarding the topological relation, as the design of indoor positioning is an environment-dependent system because of the diversity of indoor environments, the experiment must be performed in situ. Under the condition of the largest number of APs, we test only a few deployment schemes due to the strict limitation in the security region. Specifically, in the T3-C security region, Beijing Capital International Airport, there are limited Internet access ports for the positioning system, and devices that affect the normal traffic and security check of passengers are not permitted. Therefore, we deployed the maximum number of APs according to principles mentioned in Section 2.2 and changed their locations among a limited number of positions allowed by the airport side. Finally, we installed nine APs in the queue zone whose locations are depicted in Figure 5. The seven APs on the right side were deployed inside the counters, and the two on the left were deployed near the escalator. We placed metal baffle plates near the border APs on the outside of the queue zone.

### 3.2. Topological Accuracy Test

As systematic topological errors of WiFi positioning systems have a high possibility of occurrence near the border of a queue zone, we tested six points inside and three points outside the queue zone, all near the border. The results of this test are shown in Table 1; Inside-Inside means that both the test point and the positioning location were inside the queue zone; Inside-Outside means that the test point was inside the queue zone and the positioning location was outside the queue zone. We observed that 73.11% of the positioning locations had a correct topological relation when the point was inside the queue zone, and 82.79% were correct when the point was outside the queue zone. We deem these results to be sufficiently accurate for queuing time estimation and prediction [46,47].

### 3.3. WiFi-Based Estimation Model Validation

**Figure 6:**
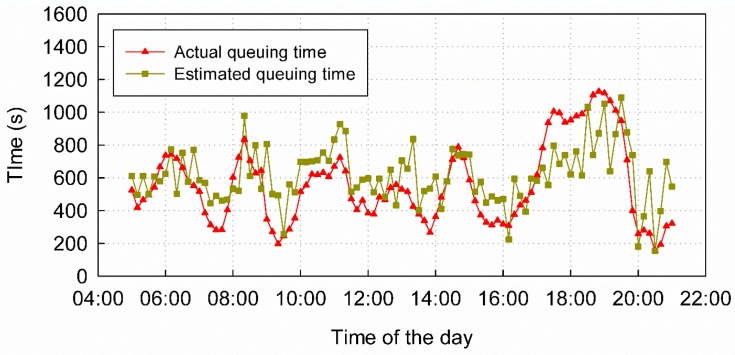
Validation results of the WiFi-based estimation model.

We collected WiFi positioning data (by RTmap Science and Technology Ltd., Beijing, China) for an entire day. The time during which queuing occurred was estimated according to the model described above. To resolve the variation in queuing time throughout the day, we divided the day into 10 min time slices. The main traffic passing through the T3-C entrance is from 5:00 a.m. to 9:00 p.m., so we only estimated the queuing time during this time-frame. We simultaneously collected check-in and check-out data from the security system and calculated the actual queuing time for validation. Figure 6 shows the validation results of our estimation. The mean absolute error of the estimation was 156 s, and 69.07% of the absolute errors were less than 200 s, which indicates that our queuing time determination and estimation model is practicable in real-life scenarios.

## 4. Case Study

### 4.1. Queuing Time Estimation for the T3-C Entrance of Beijing Capital International Airport

As positioning accuracy depends on the field environment, for the case study, we again chose the T3-C entrance of Beijing Capital International Airport to validate our methodology. Beijing Capital International Airport is the largest airport in China and the second busiest in the world, with a passenger throughput that exceeded 89 million person-time (overall number of people including reoccurring ones) in 2015. In large-scale public areas like this, queuing time affects the management and service quality. Our system can automatically detect and predict individuals’ queuing times, which is helpful for improving traffic efficiency, optimizing management, promoting service quality and improving the capability to address emergencies.

#### 4.1.1. Data Collection

We collected eight days of WiFi positioning data (by RTmap Science and Technology Ltd.) from the T3-C entrance of Beijing Capital International Airport from 11 August to 18 August. Each day included more than 6.5 million records, and the fields of these records were:

$$\left[time\_stamp,\;MAC,\;building\_ID,\;floor\_ID,\;coordinate_{X},\;coordinate_{Y},\;AP\_MAC\right]$$

where time_stamp is the time stamp recorded when a positioning location was collected; MAC is the media access control address of a mobile device; building_ID is the serial number of the building (for example, terminal T3); floor_ID denotes the floor number; $coordinate_{X}$ and $coordinate_{Y}$ are the x and y coordinates, respectively, of the location; and AP_MAC is the MAC address of the AP that provides the strongest signal for the device when positioning.

Before an individual enters the queue zone, he/she swipes their boarding pass to check-in and go through the security door on the fourth floor. Next, he/she takes the escalator downstairs to the third floor to wait for security in a queue. After the queuing process, the staff at the counter stamps the passenger’s boarding pass to check them out and the passenger leaves the queue zone to have his baggage checked. In our study, we added the time spent on escalators to the queuing time. Thus, the queuing process starts when a passenger checks in and enters the queue zone, and ends after checking out and leaving the queue zone. Therefore, the time between check-in and check-out recorded by the security system is regarded as the actual queuing time for validation.

#### 4.1.2. Data Preprocessing

**Figure 7:**
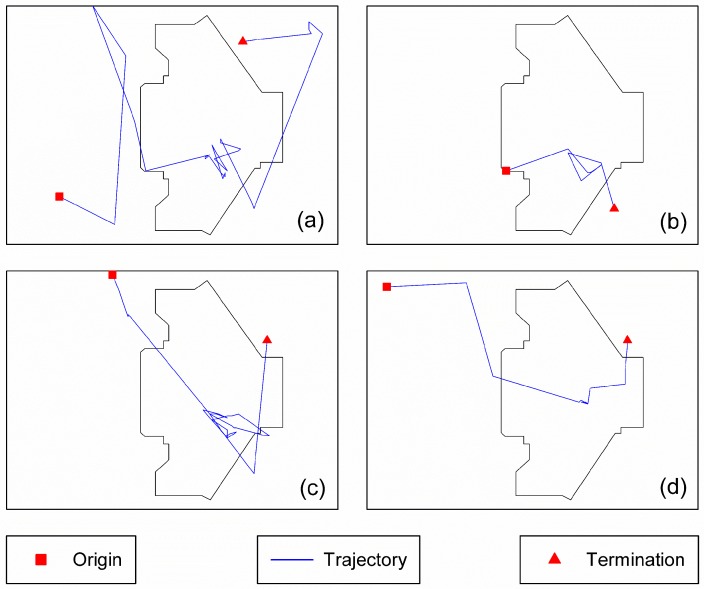
Passenger trajectory examples. (**a**–**d**) show trajectories of four different passengers in the T3-C Entrance.

In addition to the APs deployed in and around the queue zone, there are many other APs in this building that are also used to track passengers’ trajectories. Thus, the spatial extent of positioning data may capture the entire building. To remove duplicates, we marked out the research the area (an area that covers the queue zone and completed queuing trajectories), depicted as the extent of the picture frame in Figure 5, and removed records from this area based on their spatial coordinates. Before a network hardware item is produced, the manufacturer needs to apply for a MAC address from the Institute of Electrical and Electronic Engineers (IEEE). MAC addresses not found in the IEEE MAC list may be disguised MAC addresses (for security or privacy reasons) or they may be incorrect records. The records of such MAC addresses should be regarded as noise and removed. As network equipment, an AP itself can be detected by other APs. Therefore, records generated by APs also need to be removed. Furthermore, staff devices and fixed equipment that have network functions can also be detected by the positioning system, so records of such equipment should be excluded before queuing time estimation. In this study, we set 2 h as the maximum residence time in the research area. If the duration in the research area exceeds this maximum time, the MAC is regarded as the MAC of fixed equipment or a staff device, and the MAC is added to a list that is updated every day by the residence time judgment in the research area. In real-time queuing time estimation, the MAC addresses in this list are excluded. Some sample trajectories after data filtering are depicted in Figure 7.

#### 4.1.3. Parameter Calibration and Queuing Time Determination

Under the condition that there is no room for hardware deployment improvement, the algorithm plays a key role in the queuing time determination. As depicted in Figure 2, we first select a MAC occurring in a time slice and extract the trajectory of this MAC. Through topological relation judgment, we can identify the entrance and exit points of the queue. Next, as mentioned in Section 2.3.2, we judge whether the trajectory is eligible to be used for estimation according to the parameters from calibration. To ensure the integrity of the trajectory, m and n are set to 2, which means at least 2 (m) consecutive points are detected immediately before entering the queue zone, and at least 2 (n) more points are detected after leaving the queue zone. The other two parameters (k and r) are the main algorithmic factors that can be used to improve the estimation results. The calibration records of k and r are shown in Table 2, from which we can conclude that the best combination of these parameters is k=5 and r=50%; that is, at least 5 (k) points detected from the entrance to the exit point, and the proportion of points drifting out of the queue zone is less than 50% (r) from entrance to the exit point (the mean drift ratio of the positioning data used for estimation from 11 August to 18 August is less than 5% in the subsequent statistics). Finally, if the exit time of this queuing process occurs within the same time slice (the exit time of a MAC may not be in the same time slice, although it occurs in the time slice), then the passenger queuing time will be estimated from the time stamps of the trajectory.

According to field surveys, it is improbable for a passenger to queue for less than 60 s or for more than 1800 s. Therefore, after the queuing time of all passengers in a time slice has been estimated, we remove the estimated queuing times that fall outside this time range, designating them as incorrect results. Next, we remove abnormal results, which are defined as those more than or less than 1.5 standard deviations from the overall mean. Finally, we calculate the mean value of the remaining results to represent the crowds’ average queuing time in the time slice.

#### 4.1.4. Estimation Results and Validation

**Figure 8:**
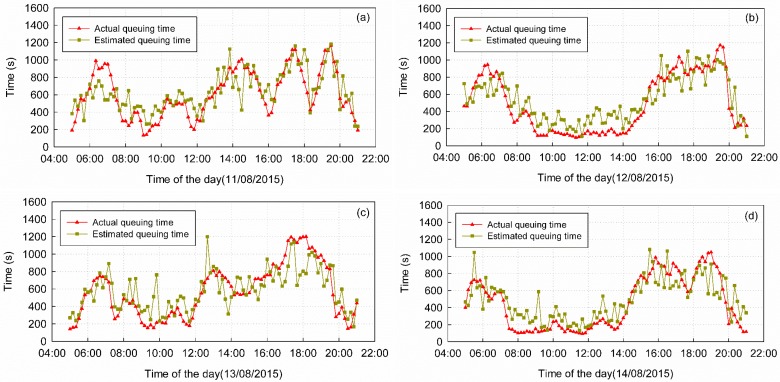
Comparison of the estimated and actual queuing times. (**a**) Comparison of the estimated and actual queuing times on 11 August; (**b**) Comparison of the estimated and actual queuing times on 12 August; (**c**) Comparison of the estimated and actual queuing times on 13 August; (**d**) Comparison of the estimated and actual queuing times on 14 August.

We estimated queuing time in 10 min intervals between the hours of 5:00 a.m. to 9:00 p.m. from 11 August to 18 August. For validation, the corresponding check-in and check-out data from the airport security system, which we refer to as security data, were collected as the ground truth. These data record passenger IDs, check-in and check-out times, and number of security counters. By collating these records according to passenger ID, we calculated every passenger’s queuing time. The queue zone has four special channels (Figure 5, top and bottom corners of the queue zone), which are prepared for “hurry-off” passengers (channels 1 and 2) and VIP passengers (channels 25 and 26). Passengers leaving from these channels do not need to queue like the other passengers. Therefore, the queuing time of passengers who pass through the queue zone via these channels is usually short, and the integrated trajectories of these passengers can scarcely be detected by the positioning system. In accordance with the positioning data results, the security records of these channels are removed and the actual queuing times determined from security data that are less than 60 s or more than 1800 s are also removed as incorrect results. The actual queuing times are aggregated into 10 min time slices according to the check-out time stamps. The mean queuing time calculated from security data in the same time slice can validate the results estimated by the WiFi positioning data (Figure 8).

The statistical characteristics of the estimation errors are listed in Table 3. The mean queuing time from 11 August to 18 August was 546 s, while the estimated mean queuing time was 570 s. The mean absolute error (MAE) of the estimation results was 147 s, approximately 26.92% of the actual queuing time. On average, 25.39% of the absolute errors (AEs) were less than 60 s, 49.61% were less than 120 s, 69.20% were less than 180 s and 73.58% were less than 200 s.

### 4.2. Queuing Time Prediction for the T3-C Entrance

Based on the estimation results, we can predict future queuing time using the three prediction models presented in Section 2.4. For NAR(p), the coefficients of the prediction models are determined by the least squares method according to previous estimation results and the actual queuing times. To determine the order parameter p, i.e., the number of independent variables required to build the prediction model, we tested models based on one independent variable ($W_{t-10}$, i.e., p=1) and two independent variables ($W_{t-10}$ and $W_{t-20}$, i.e., p=2). The error statistics for both models are summarized in Table 4. Experiments 1 and 2 use the same model training data and sample data for their predictions. Likewise, experiments 3 and 4 have the same model training and prediction data. Based on these experiments, we conclude that p=2 is an appropriate order parameter for the NAR(p) model.

Regarding the regression coefficients ($\varphi_{i}$ in Equation (3)), we need to determine how many days of training data are suitable for NAR(2). We tested three solutions, in which NAR(2) was trained by the estimation results and ground truth of the preceding one, two or three days. Then, we used NAR(2) trained by these solutions to predict the queuing times for the same days. Table 5 shows the mean error statistics of the prediction results, from which we conclude that NAR(2) trained with the estimation results and ground truth of the previous three days (for example, using the model trained by the results of 11–13 August to predict the queuing time on 14 August) are theoretically sound. 

For real-time queuing time prediction, the step length of time slices for prediction is set to 1 for the Holt-Winters and STL-ARIMA models, that is, we only use these models to predict queuing time in the upcoming time slice. The periodic parameter of the models (Winters and STL-ARIMA) is set to 97 (97 time slices per day from 5:00 a.m. to 9:00 p.m.). The parameters of NAR(2) are calculated using the least squares method. The Holt-Winters and STL-ARIMA predictions are implemented using the forecast package [48] in R Language [49], and the remaining parameters, except the time step and period, are automatically tuned by the procedure. The average prediction errors of these three models from 11 August to 14 August are shown in Table 6, which shows that NAR(2) achieves better results than the other two models.

**Figure 9:**
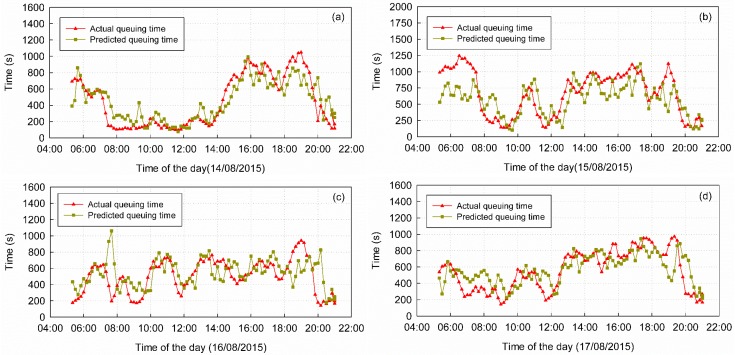
Comparison of the predicted and actual queuing times. (**a**) Comparison of the predicted and actual queuing times on 14 August; (**b**) Comparison of the predicted and actual queuing times on 15 August; (**c**) Comparison of the predicted and actual queuing times on 16 August; (**d**) Comparison of the predicted and actual queuing times on 17 August.

Some prediction results from the NAR(2) model are plotted in Figure 9, along with the actual queuing times. The mean queuing time from 14 August to 18 August was 542 s, while the mean queuing time predicted by NAR(2) was 548 s. The MAE of the prediction results was 149 s, which is approximately 27.49% of the actual queuing time. As shown in Table 6, on average, 28.21% of the AEs were less than 60 s, 50.53% were less than 120 s, 68.42% were less than 180 s and 73.05% were less than 200 s. However, the results also show a few weaknesses, for example, the beginning of the prediction curve in Figure 9b and the jump point in Figure 9c.

## 5. Discussion

The main goal of this study was to develop a method to predict queuing time based on WiFi positioning data in indoor scenario. The results of a case study at Beijing Capital International Airport show that the method we proposed is feasible for real-time queuing time prediction. In contrast to WiFi-based queuing monitoring systems such as LineKing [15,22] and Wang’s method [23,24,25], and systems based on other sensors mentioned in Section 1, our method does not require users to install specific apps on their mobile devices, which means it is a completely passive queue monitoring method and it can be easily extended to new applications. In addition, in the case study, the estimation and prediction error of our method were approximately 26.92% and 27.49%, respectively, while the estimation error of LineKing is approximately 24%–36% in a coffee shop. Wang’s method had an error of approximately 10 seconds. However, because Wang et al. only used one WiFi monitor, it is difficult for their method to address long or disordered queues using a signal strength threshold parameter to distinguish the starting time of a queuing process. As our method was only tested in a security region with strict limitation and because it is scenario-dependent, the comparisons can only be viewed as a reference. To compare the prediction accuracy at different time periods, we aggregated the time slices of a day into 3 slices (i.e., 5:00 a.m.–12:00 p.m., 12:00 p.m.–5:00 p.m. and 5:00 p.m.–9:00 p.m.). The MAEs of the prediction results were 36.12% from 5:00 a.m. to 12:00 p.m., 18.49% from 12:00 p.m. to 5:00 p.m. and 28.68% from 5:00 p.m. to 9:00 p.m. Because of the traffic rush in the morning, the high variation in traffic may lead to large errors in the period 5:00 a.m.–12:00 p.m. In addition, the environmental factors on the morning of 15 August, which will be explained in the following section, affected the accuracy of this period. Comparison of the prediction results from different models indicates that our NAR model has better accuracy than the other two models. There are two possible reasons for this phenomenon: (1) the NAR model can be trained using historical ground truth, while the other two models only consider the observations; and (2) the Holt-Winters and STL-ARIMA models are good at addressing sequences with regular cycles, while the queuing time pattern in our research varies by days, especially between weekdays and weekends.

During the deployment phase of our positioning system, we made some arrangements for the original APs at the T3-C Entrance. Two main elements were concerned, signal coverage and signal overlap. Because the signal transmitting range is approximately 50 m to 100 m, which exceeds the radius of the queue zone, APs deployed near the border of the queue zone can ensure the integrity of signal coverage in the queue zone. Metal baffle plates were placed to reduce signal overlap between the inside and outside of the queue zone, which may cause drift phenomenon and lead to topological errors. Thus, when users leave the queue zone, they receive a weak signal from the APs inside the queue zone due to the metal baffle plates. However, as the distance from the APs in surrounding shops to the queue zone is no more than 30 m, normal Internet service is barely affected.

There are two types of factors affecting the accuracy of the results: environmental factors and systematic factors. Environmental factors affect the normal queue and security check, and often lead to outliers, for example, the large errors in the prediction results, especially 5:00 a.m.–8:00 a.m., 15 August in Figure 9b and 7:00 a.m.–8:00 a.m., 16 August in Figure 9c. Historical weather records indicated that, on 15 August, 22 out of 34 municipalities and capital cities experienced rain, which may have stranded passengers due to flight delays and led to the error from 5:00 a.m. to 8:00 a.m. in Figure 9b. Through discussions with the airport staff, we concluded that other environmental factors that can generate outliers include sudden traffic variation, security strategy adjustment (for example, security gate number change) and large traffic flow along the outside of the queue zone near the boundary. Because we do not have additional information about this time period, we cannot give the exact reason. Nevertheless, the existence of outliers is due to one or more of the above reasons. System factors include topological accuracy, the packet delivery frequency of mobile devices, and lags in the models.

### 5.1. Topological Accuracy

Although we took a series of measures to ensure the topological accuracy during the deployment of the APs and proposed a parameter called *drift ratio* to alleviate the impact of drift phenomenon, topological accuracy remains a problem that adversely affects queuing time estimation. The topological accuracy of our positioning system in this research may be affected by the deployment of APs, the positioning method, and the hardware status.

#### 5.1.1. Deployment of APs

The number and locations of APs are restricted by the site environment and the limited number of Internet access ports at the T3-C entrance of Beijing Capital International Airport. Furthermore, facility transformation and maintenance require extra investment. Therefore, additional hardware facility installations are not accepted by the airport authority, and the optimal deployment scheme cannot be realized, which affects the topological accuracy. For example, in this study, the two APs deployed on the left in Figure 5 were not in the optimal locations.

#### 5.1.2. Positioning Method

For WiFi positioning, fingerprinting usually yields better accuracy and stability than trilateration. However, fingerprinting requires many signal measurement during the deployment phase, and when AP deployment changes, this measurement needs to be repeated. Therefore, to minimize the effects on the normal traffic and security management of the airport, we chose the trilateration method, which is simple but has relatively poor accuracy and stability and thus may reduce the topological correctness.

#### 5.1.3. Hardware Status

For unknown reasons, the AP equipment may power off, disconnect from the Internet or break down. Furthermore, different APs of the same brand and version may have different signal propagation characteristics, and the signal of an individual AP may be unstable over time. These factors may affect the topological correctness and thus the estimation accuracy, for example, the jump points in Figure 8c,d.

### 5.2. Packet Delivery Frequency of Mobile Devices

As our proposed queuing time solution does not require that individuals install an app on their mobile devices, the packet delivery frequency only depends on the status of their devices. If individuals connect their mobile devices to the APs and continuously use the WiFi network, then their devices will deliver packets regularly and at a high frequency. Thus, these trajectories are dense with sample points, and the resulting estimations have high accuracy. However, if an individual keeps the WiFi module on but does not connect to APs or connects to APs but does not use the device continuously during the queuing process, the sample points on the trajectory may be sparse. The sparsity of sample points may lead to estimation errors.

### 5.3. Lags in the Models

As all models in our research use previous queuing estimations for queuing time prediction, the predictions lag behind the actual queuing time, especially in moments when the queuing time increases or decreases sharply, which may occur when there is large variation in the number of people queuing over a short time interval, for example, the sequence near the end in Figure 9c,d. We observe this phenomenon in the estimation results of this study. Although our regression model corrects some lag deviation, it is not eliminated completely. Moreover, correlations between previous estimation results and future queuing times may vary in time, which may affect the reliability of the queuing time predictions.

## 6. Conclusions and Future Work

In this paper, we presented an overview of previous queuing time monitoring techniques and solutions, and proposed a novel methodological framework to estimate and predict queuing time based on WiFi positioning data in indoor scenarios. Our method is composed of hardware deployment and the algorithm. In the hardware deployment part, we deployed APs on the border of a queue zone and installed metal baffle plates for the border APs to improve the topological correctness. In the algorithmic part, as mentioned in Section 2.3, to reduce the error caused by the “drift phenomenon” of WiFi positioning and the impact of trajectory point sparsity, we proposed four parameters (m, n, k, and r) to identify relatively reliable samples as representatives of queuing time in specific time slices. In different application scenarios, these parameters must be calibrated before the method is used for queuing time estimation and prediction. In the case study at the T3-C entrance of Beijing Capital International Airport, although the number and location of APs were restricted by limited internet access ports and deployment locations, the minimum MAE of the estimation results from 11 August to 18 August was 147 s, which is 26.92% of the average actual queuing time. In contrast with the results of previous WiFi-based queuing time estimation methods relying on mobile apps, the accuracy of our simple but carefully designed method is within the acceptable range.

For prediction, we proposed the NAR model. The main difference of NAR with respect to general AR model is that NAR is trained using previous observations and the ground truth. To determine the order of the NAR model and the number of days’ results for model training, we conducted a series of experiments. The results showed that an order of 2 and number of previous days of 3 produced sufficiently accurate predictions. Using this model, we predicted the queuing time in T3-C entrance from 14 August to 18 August in real time. The minimum MAE of the predictions came from the NAR model, i.e., 27.49% of the average actual queuing time, which was better than the Holt–Winters and STL-ARIMA models. 

Our research shows that indoor queuing time can be estimated and predicted using WiFi positioning data. Compared with previous studies, our method has four advantages: (i) it is robust to positioning accuracy when topological correctness is reliable; (ii) it does not require special sensors to cope with the widespread nature of WiFi APs; (iii) it is completely passive because it does not require individuals active participation or for them to install an app on their mobile devices; and (iv) it does not need to consider the shape of a queue, only the individuals’ spatiotemporal location and the extent of the queue zone. As WiFi is widely used in public areas, our method is applicable in locations such as train stations, museums and supermarkets. However, it can only be used in places where there are queue zones with well-defined borders. In actual application scenarios, our method is restricted by the site environment and the drift of the WiFi positioning system. Therefore, the accuracy of estimation and prediction results may not be fully satisfactory. In future work, a positioning algorithm with better location accuracy and stability will be used to improve the topological accuracy. For example, fingerprinting could be used to replace triangulation, and the deployment strategy could be improved to reduce the impact of the indoor environment. Additionally, technical and algorithmic solutions will be explored to alleviate the impact of drift.

## Figures and Tables

**Table 1:** Results of the topological test near the border of the queue zone.

	Inside	Outside
Inside	73.11%	26.89%
Outside	17.21%	82.79%

**Table 2:** Error statistics of different parameter combinations.

Parameter Combinations		Error Statistics						
k	r	Mean	Std.	MAE	AE ≤ 60 s	AE ≤ 120 s	AE ≤ 180 s	AE ≤ 200 s
5	50%	30.27	177.30	144.57	32.99%	49.48%	**71.13%**	**76.29%**
6	50%	38.46	189.37	146.60	30.93%	50.52%	69.07%	**73.20%**
7	50%	49.39	193.98	151.24	27.84%	50.52%	69.07%	**72.16%**
8	50%	52.08	193.59	151.72	26.80%	52.58%	69.07%	**73.20%**
9	50%	61.11	195.71	157.38	25.77%	52.58%	65.98%	**71.13%**
10	50%	63.47	193.69	162.19	22.68%	47.42%	62.89%	69.07%
5	40%	30.10	196.18	148.54	34.02%	52.58%	67.01%	**72.16%**
5	30%	24.36	207.67	158.72	29.90%	49.48%	65.98%	**70.10%**
5	20%	15.21	210.29	160.19	27.84%	52.58%	67.01%	69.07%
5	10%	−2.76	216.68	167.48	24.74%	48.45%	65.98%	69.07%
5	0%	−49.45	228.15	183.47	21.65%	42.27%	59.79%	61.86%
6	40%	35.54	195.21	150.07	29.90%	49.48%	67.01%	**72.16%**
6	30%	31.21	207.15	160.91	26.80%	47.42%	64.95%	**70.10%**
6	20%	20.18	209.47	161.04	26.80%	50.52%	67.01%	**70.10%**
6	10%	1.17	215.61	166.61	24.74%	49.48%	65.98%	69.07%
6	0%	−40.43	230.45	183.76	21.65%	41.24%	58.76%	60.82%
7	40%	47.71	200.10	155.34	27.84%	49.48%	65.98%	69.07%
7	30%	42.49	212.83	165.82	25.77%	46.39%	63.92%	68.04%
7	20%	38.49	215.77	167.98	27.84%	46.39%	64.95%	67.01%
7	10%	21.25	229.22	174.12	25.77%	47.42%	63.92%	68.04%
7	0%	−21.29	250.22	194.09	23.71%	41.23%	56.70%	61.86%
8	40%	51.51	201.10	156.96	26.80%	50.52%	67.01%	69.07%
8	30%	46.30	214.06	167.07	25.77%	47.42%	64.95%	67.01%
8	20%	42.15	218.55	172.81	24.74%	46.39%	62.89%	65.98%
8	10%	23.67	230.53	175.55	27.84%	45.36%	62.89%	68.04%
8	0%	−18.15	251.78	195.19	23.71%	39.18%	56.705	61.86%

Note: Values exceeding 70% are emphasize using boldface. Std.: standard deviation, MAE: mean absolute error, AE: absolute error.

**Table 3:** Queuing time estimation errors from 11 August to 18 August.

Date	Mean	Std.	MAE	AE ≤ 60 s	AE ≤ 120 s	AE ≤ 180 s	AE ≤ 200 s
11 August	30.27	177.30	137.14	32.99%	49.48%	71.13%	76.29%
12 August	54.80	167.90	147.97	19.59%	45.36%	64.95%	71.13%
13 August	16.52	189.23	143.07	28.87%	51.55%	69.07%	74.23%
14 August	44.25	175.83	147.38	23.71%	45.36%	67.01%	70.10%
15 August	−66.91	247.02	197.20	21.65%	37.11%	58.76%	61.86%
16 August	40.12	186.46	142.63	24.74%	57.73%	77.32%	78.35%
17 August	16.53	163.20	133.65	23.71%	49.48%	72.16%	77.32%
18 August	58.41	143.63	123.12	27.84%	60.82%	73.20%	79.38%

Std.: standard deviation, MAE: mean absolute error, AE: absolute error.

**Table 4:** Comparison of the prediction errors from NAR(1) and NAR(2).

Error Statistics of Prediction Results from Model NAR(1)					Error Statistics of Prediction Results from Model NAR(2)				
No.	Mean	Std.	MAE	AE ≤ 200 s	No.	Mean	Std.	MAE	AE ≤ 200 s
01	46.55	195.15	177.05	60.00%	02	34.24	178.60	155.15	66.32%
03	−38.89	216.97	164.80	69.47%	04	−35.40	203.05	161.41	69.47%
05	41.51	190.07	160.52	69.47%	06	27.38	163.27	132.29	77.89%

Std.: standard deviation, MAE: mean absolute error, AE: absolute error. W_t−10_: average queuing time from time t−10 min to t; W_t−20_: average queuing time from time t−20 min to t−10 min.

**Table 5:** Comparison of the prediction errors from NAR(2) trained using different data.

Models	Prediction Error Statistics			
	Mean	Std.	MAE	AE ≤ 200 s
Models trained by estimation results of previous 1 day	13.19	219.23	168.50	69.47%
Models trained by estimation results of previous 2 days	−10.53	214.29	161.36	70.52%
Models trained by estimation results of previous 3 days	−14.41	213.72	160.47	70.88%

Note: The independent variables of these prediction models are $W_{t-10}$ and $W_{t-20}$. Std.: standard deviation, MAE: mean absolute error, AE: absolute error.

**Table 6:** Queuing time prediction errors of different models.

Model	Mean	Std.	MAE	AE ≤ 60 s	AE ≤ 120 s	AE ≤ 180 s	AE ≤ 200 s
NAR(2)	5.21	188.33	148.95	28.21%	50.53%	68.42%	73.05%
**Holt-Winters**	19.06	206.89	162.42	23.71%	44.74%	64.54%	67.84%
**STL-ARIMA**	18.15	188.47	152.90	24.33%	45.98%	64.12%	69.28%
